# The Optic Nerve at Stake: Update on Environmental Factors Modulating Expression of Leber’s Hereditary Optic Neuropathy

**DOI:** 10.3390/biomedicines12030584

**Published:** 2024-03-06

**Authors:** Pierre Layrolle, Christophe Orssaud, Maryse Leleu, Pierre Payoux, Stéphane Chavanas

**Affiliations:** 1 Toulouse NeuroImaging Center (ToNIC), INSERM/Toulouse University UMR 1214, CHU Toulouse Purpan, 31024 Toulouse, France; 2 Assistance Publique-Hôpitaux de Paris (AP-HP), CRMR Ophtara, Paris-Cité University, 20 Rue Leblanc, 75015 Paris, France; 3 Association Ouvrir Les Yeux, 45 rue François Gauthier, 62300 Lens, France

**Keywords:** Leber’s hereditary optic neuropathy, optic neuropathy, environmental factors, neurodegeneration, mitochondrial disease, drugs

## Abstract

Optic neuropathies are characterized by the degeneration of the optic nerves and represent a considerable individual and societal burden. Notably, Leber’s hereditary optic neuropathy (LHON) is a devastating vision disease caused by mitochondrial gene mutations that hinder oxidative phosphorylation and increase oxidative stress, leading to the loss of retinal ganglion neurons and axons. Loss of vision is rapid and severe, predominantly in young adults. Penetrance is incomplete, and the time of onset is unpredictable. Recent findings revealed that the incidence of genetic LHON susceptibility is around 1 in 1000, much higher than believed till now. Environmental factors are critical in LHON triggering or severity. Families at risk have a very strong demand for how to prevent the onset or limit the severity of the disease. Here, we review recent knowledge of the extrinsic determinants of LHON expression, including lifestyle, dietary supplements, common chemicals, and drugs.

## 1. Introduction

**Figure 1:**
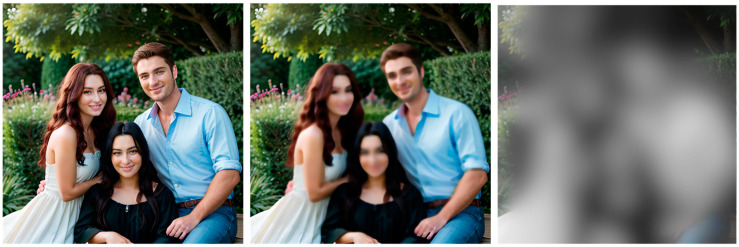
Graphic representation of visual impairment in LHON. Front view photograph of three people was generated with artificial intelligence using the AI image generator from MyEdit (myedit.online) (**left**), submitted to patients, and then processed by the authors according to the indications of the patients to best represent how they perceive the original photograph. Shown are illustrations of visual perception after the recent onset of LHON (**middle**) or in chronic LHON (**right**). Note the spared peripheric zones (**middle**,**right**).

Optic neuropathy (ON) refers to loss of vision caused by degeneration of the optic nerve. Syndromic or non-syndromic optic neuropathies are a considerable burden, manifesting in disability and the associated comorbidities, loss of employability, and medical, societal, and educational care. ON occurs in a broad spectrum of inherited or acquired diseases of various etiologies. ON pathogeny may arise from ischemic [1], traumatic [2], toxic or nutritional [3] causes. It can be of complex etiology since it underlies vision loss in glaucoma (the fourth most common cause of blindness with 4 million cases worldwide) [4] and can be associated with Alzheimer’s or Parkinson’s diseases [5]. Lastly, ON can result from gene mutations causing mitochondrial dysfunction in the hereditary optic neuropathies (HON) group of diseases that covers dominant, recessive, or X-linked inherited optic atrophies and Leber’s hereditary optic neuropathy (LHON, OMIM #535000) [6]. LHON is caused by point mutations within the mitochondrial (mt) genome and, therefore, is inherited maternally. LHON is a clinical entity that is distinct from Leber’s hereditary optic neuropathy autosomal recessive (LHONAR, OMIM #619382) caused by nuclear gene mutations [7]. In many cases, LHON presents with loss of vision due to optic nerve injury as the single outcome and, therefore, can be considered to be a paradigm for optic neuropathy. When LHON is triggered, the loss of vision is rapid (a few weeks to months), severe (predominantly below the legal threshold for blindness), initially unilateral, and then bilateral. LHON patients present with an impaired visual field with a central scotoma of variable severity and dyschromatopsia, whereas peripheral vision can be spared to some extent (Figure 1).

When the first eye is affected, the contralateral eye may compensate, delaying detection of the disease. Diagnosis odyssey is frequent. Visual acuity stabilizes within the first year after onset, initiating the lifelong chronic phase of LHON. Satisfactory therapeutic drugs are still needed, as the only authorized treatment (idebenone) provides variable and limited improvement of vision in only half of the patients treated [8]. Gene therapy based on an AAV2 pseudoviral vector has shown promising but inconclusive results to date, and the corresponding marketing authorization application to the European Medicines Agency has recently been withdrawn [9,10]. Estimates of LHON prevalence range from 1 in 30,000 to 1 in 50,000 in Europe [11], 1 in 50,000 in Japan [12], and 1 in 60,000 to 1 in 68,000 in Australia [13,14]. LHON penetrance is incomplete and has been estimated in the range of 18–50% in males and 4–32% in females [14,15]. These findings highlight the importance of secondary factors modulating LHON expression. Environmental factors are critical in triggering LHON and in its severity. Notably, tobacco smoking and alcohol abuse have been associated with LHON for years [16]. A series of articles have documented other triggers, such as the intake of therapeutic drugs unrelated to LHON or environmental smoke or, conversely, candidate molecules possibly beneficial to patients or asymptomatic carriers. Previously published reviews, however, have not always encompassed the full diversity of extrinsic factors. In addition, important recent findings based on large-scale genome studies in Australia and the United Kingdom revealed that the prevalence of LHON primary mutations in the general population was 1 in 800–1000 [13,17]. These figures are consistent with a previous assessment of 1 in 300 in a population northeast of the United Kingdom [18] and are much greater than estimated in previous years (predominantly around 1 in 10,000). Therefore, the population with LHON susceptibility is probably much larger than has been thought for years.

Families at risk have high expectations for information, particularly in the context of growing awareness of the effects of lifestyle on health. It is, therefore, of utmost importance to provide them with recommendations on how best to avoid triggering and/or limit the severity of LHON. In this review, we propose a summary of the pathophysiology of LHON and a wide-ranging update of the extrinsic determinants of LHON expression.

## 2. Neurodegeneration Pattern

**Figure 2:**
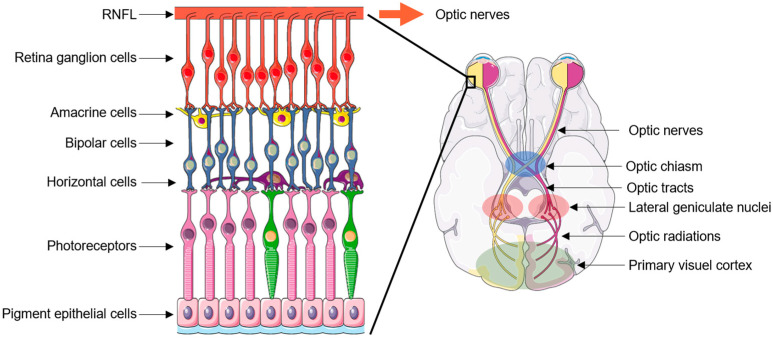
Graphic representation of retina cell organization (**left**) and of the optic pathways (**right**). The yellow and purple pathways correspond to the left and right visual fields, respectively. RNFL: retinal neural fiber layer.

At the forefront of the central nervous system, the retina lines the inner back of the eye and contains a wealth of specialized cells of epithelial, glial, and neuronal nature, intricately connected in a stratified pattern and which together convert light into biological, then electrophysiological signals [19]. The output cells of the retina are specialized neurons named retinal ganglion cells (RGCs) (Figure 2).

RGCs lie at the outer layer of the retina from which they project their axons. The axons of the RGCs collectively form the retinal neural fiber layer (RNFL), which is up to 100 µm thick and found on the surface of the retina, then cross the so-called lamina criblosa to ultimately form the optic nerves [19]. The optic nerves from both eyes meet at the optic chiasm, where their fibers decussate to form the optic tracts. The optic tracts synapse in the lateral geniculate nuclei (LGN) of the thalamus. Axons from the LGN then form the optic radiations connected to the visual cortex [20] (Figure 2).

In LHON, RGC and axon loss involve preferentially the small fibers of the array that subserve central vision, namely the papillomacular bundle [21]. The optic disc becomes pale, first temporally and then totally, due to axon loss. As shown by optical coherence tomography (OCT), in LHON patients, the RNFL initially swells during the first months before decreasing severely and stabilizing in the chronic phase [21]. Histopathological examination of sections from deceased LHON patients reported extensive loss of RGCs and severe thinning of the RNFL, and, at the optic nerve level, variably reduced temporal and central fiber populations and reduced nerve cross-sectional area (up to 95% and 40%, respectively, as compared to controls), along with fibrocytic scarring and degeneration dust [22,23,24]. It has been suggested that degeneration could persist throughout the patient’s life, even long after visual function has stabilized [24].

MRI studies revealed several white matter abnormalities, all indicative of cell loss, demyelination, and/or axonal death in stable LHON patients: reduced volume of the optic nerves, chiasm, tracts, LGN, and visual cortex, along with reduced patterns of magnetization transfer ratio and fractional anisotropy, and increased diffusivities in the visual pathways, as compared to healthy controls [25]. MRI exploration of acute LHON patients (i.e., soon after triggering of the disease) revealed chiasm enlargement and T2 hyperintensities in the optic nerves, which may result from axonal loss, demyelination, and/or gliosis [19,25].

In rare cases, multiple sclerosis (MS)-like symptoms may arise in LHON patients (Harding’s disease), and as many as 25% of a cohort of 31 LHON patients were found to exhibit T2 hyperintensities similar to those found in MS [26]. The possibility of overlapping neuropathogenic events in LHON and multiple sclerosis remains, however, uncertain.

## 3. Genetics and Pathophysiology

**Figure 3:**
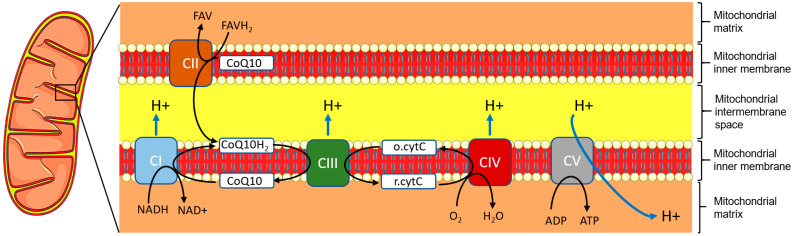
Graphic summary of the ETC and OXPHOS. The five protein complexes (CI–V) are embedded in the inner mitochondrial membrane. Complex I and complex II catalyze the reduction of ubiquinone (CoQ10) to ubiquinol (CoQ10H_2_) upon oxidation of nicotinamide adenine dinucleotide (NADH/NAD+) or flavin adenine dinucleotide (FADH_2_/FAV), respectively. Complex III catalyzes the reduction of cytochrome c (o.cytC) upon oxidation of CoQ10H_2_. Complex IV catalyzes the reduction of dioxygen to water upon oxidation of reduced cytochrome c (r.cytC). Protons are released into the intermembrane space at each step and are driven back into the mitochondrial matrix through complex V, which generates ATP from ADP.

In 95% of cases, LHON is caused by one of only three primary mitochondrial gene mutations: m.3460G>A (A52T), m.11778G>A (R340H) and m.14484T>C (M64V) in the genes encoding the nicotinamide adenine dinucleotide (NADH): ubiquinone dehydrogenase subunits (ND-) 1, 4, or 6, respectively. These mutations are found at the homeoplasmic state in most cases, i.e., all copies of the mtDNA are identical and harbor the mutation. These three mutations, as with the other, rarer LHON mutations, result in dysfunction of NADH:ubiquinone dehydrogenase [15]. NADH:ubiquinone dehydrogenase, most often referred to as complex I, is the first of the five enzymatic multiprotein complexes forming the electron transport chain (ETC) embedded within the inner mitochondrial membrane. The ETC supports oxidative phosphorylation (OXPHOS), which generates ATP after a cascade of oxidation-reduction reactions between the five complexes and cofactors such as ubiquinone, also known as coenzyme Q10 (CoQ10) and cytochrome c [27] (Figure 3).

First, complex I catalyzes the oxidation of NADH and reduction of CoQ10 to CoQ10H_2_ (ubiquinol). Complex II works in parallel to complex I: as the latter transfers two electrons from NADH to CoQ10, complex II transfers two electrons from flavin adenine dinucleotide (FADH_2_) to CoQ10. CoQ10H_2_, as an electron donor, is in turn oxidized back to CoQ10 by complex III upon reduction of cytochrome c. Finally, electrons from cytochrome c are transferred to dioxygen to reduce it to water upon catalysis by complex IV. Throughout this entire process, a proton gradient is generated from the mitochondrial matrix to the mitochondrial intermembrane space. This proton gradient allows complex V (ATP synthase) to catalyze the production of ATP from ADP, providing energy to the cell. It is important to note that the dynamic balance between the oxidized and reduced forms of CoQ10 is critical for regulating reactive oxidative species (ROS) levels and oxidative stress. A wealth of studies with animal or cell models or patient tissues have shown that dysfunction of the ETC because of LHON mutations results in decreased ATP production, increased oxidative stress, mitochondrial apoptosis, and, in turn, cell death [21]. Neurons are particularly sensitive to oxidative stress for multiple molecular and cellular reasons that are not in the scope of the present review and have been reviewed elsewhere recently [28]. Notably, two peculiar features of RGCs make them both extremely energy-demanding and fragile neurons: their axons are bent at the end proximal to the cell body, and they are not myelinated before they exit the retina through the lamina criblosa. Experimental and recent clinical evidence indicates the prevailing role of increased oxidative stress in the neuropathogeny of LHON [28,29].

## 4. Environmental Protective Factors

### 4.1. Diet

Nutritional optic neuropathies are not in the scope of the present article, but they highlight the importance of proper uptake of nutrients for the health of the optic nerves [3]. A healthy, balanced diet is obviously essential for LHON patients or healthy carriers of an LHON mutation (both of which, for concision, we will refer to as “LHON carriers” when not distinctive). It is well-established that the high-fat “Western” diet has harmful outcomes on health, particularly on mitochondrial function [30]. Conversely, specific diets, namely the ketogenic and Mediterranean diets, may be beneficial for preventing or delaying the onset or limiting the severity of LHON.

The ketogenic diet is based predominantly on the consumption of fats, with the minimized intake of carbohydrates, so that energy is obtained from ketone bodies generated from the oxidation of fatty acids (β-hydroxybutyrate, acetoacetate, and acetone) rather than from glucose [31]. Several in vitro, preclinical and clinical studies have revealed an impressive array of benefits from ketone bodies, readily used as an efficient source of energy by neurons, along with the ability to reduce demyelination, axonal degeneration, ROS levels, increase the levels of antioxidants, enhance mitochondrial biogenesis and the activity of related transcription factors (peroxisome proliferator-activated receptor [PPAR] γ, hypoxia-inducible factor [HIF]-1α), decrease the levels of inflammatory cytokines (Tumor Necrosis Factor [TNF] α, interleukins 1-β, 2, 4, 6) or effectors (nuclear factor [NF]-κB, inducible nitric oxide synthase [iNOS]), and provide clinical benefits to patients with Alzheimer’s or Parkinson’s diseases or MS [31,32]. For these reasons, the ketogenic diet has been proposed as a potential means of limiting LHON progression or severity [33]. Consistent with this hypothesis, a recent study revealed that the ketogenic diet improved RGC survival and function in a mouse model of traumatic ON [34]. Nevertheless, the outcomes of the ketogenic diet on mitochondrial dynamics and mitophagy remain contradictory [30]. Furthermore, in the rare LHON patients with heteroplasmic mutations, the ketogenic diet may alter mtDNA content through selective pressure and clonal selection of mitochondria, possibly exacerbating cell death and visual loss [33,35]. The ketogenic diet modulates and reshapes gut microbiota [36] and, therefore, may impact neurological health positively or negatively through the brain-gut axis, especially with respect to oxidative stress [37]. Lastly, long-term compliance with this restrictive diet remains an issue. The Mediterranean diet is far less restrictive than the ketogenic diet and is characterized by a predominance of vegetables, fruits, nuts, grains, fish, seafood, and olive oil (including for cooking), low consumption of red wine, and limited consumption of meat and refined sugars [38]. Although it also considerably limits glucose intake, the Mediterranean diet does not rely as much on a metabolic effect mimicking fasting as the ketogenic diet but rather on the supply of abundant molecules that are beneficial to cellular homeostasis and mitochondrial function, such as polyphenols and unsaturated fatty acids [30]. It is assumed that the high abundance of strong antioxidant molecules in Mediterranean plants is the result of natural selection over time in this region that is highly exposed to ultraviolet radiation from sunlight [39]. The Mediterranean diet provides well-established neuroprotective, immunomodulatory, and antioxidant properties, is associated with a lower risk of Alzheimer’s and Parkinson’s diseases, and is assumed to maintain a favorable gut microbiote (eubiosis) through the carbohydrates from plant fibers [40]. Recent works have highlighted that the Mediterranean diet, or some aspects of it, composed of nutrients (such as ligstroside, hydroxytyrosol, oleocanthal and oleuropein from olive oil or allicin from garlic), is beneficial to mitochondria by reducing ROS production and availability, improving survival, biogenesis, and OXPHOS [39,41,42,43,44]. Together, these findings sound particularly significant in the context of mitochondrial cytopathies with abnormal OXPHOS such as LHON.

### 4.2. Food Supplements or Inputs

Dietary supplements are extracts, concentrates, or combinations of vitamins, minerals, amino acids, or essential fatty acids intended to restore or maintain an adequate intake of nutrients but not to treat or prevent diseases [45]. A growing number of dietary supplements are being marketed and advertised with varying degrees of thoroughness. The outcomes of several dietary supplements in glaucoma were recently reviewed and provide some insight into their putative role in ONs.

Glaucoma is a frequent optic neuropathy with intraocular pressure (IOP) as the leading risk factor, although some cases do not present with elevated IOP. Vascular dysregulation, immune-inflammatory response, excitotoxicity, oxidative stress, and/or mitochondrial dysfunction may underly glaucoma [45]. Apart from those whose action is related to IOP, vascular dynamics, or non-neural retina cells, some supplements were found to have beneficial outcomes on mitochondrial function and/or RGC survival in mouse models: vitamin B3 (nicotinamide), forskolin, Ginkgo biloba extracts (EGb 761) and Erigeron breviscapus hand-mazz (EBHM, from a traditional Chinese herb). EGb 761 and EBHM were also reported to improve the visual field in patients with non-IOP-dependent glaucoma and in patients with glaucoma in the absence of any detectable change in IOP in either treated or placebo groups [45].

**Figure 4:**
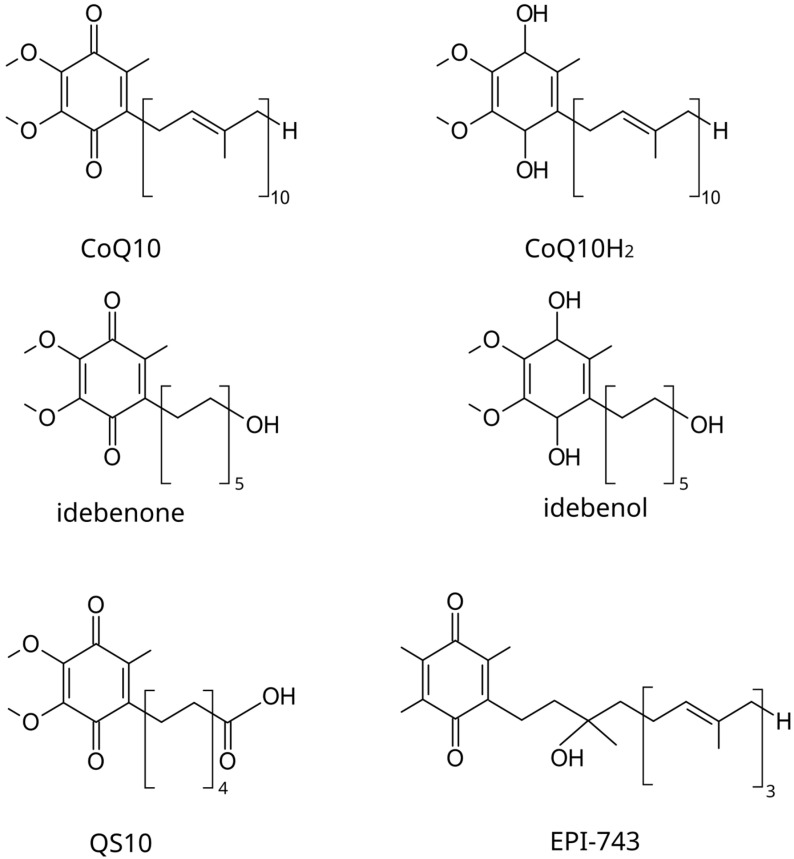
Molecular structures of the benzoquinones CoQ10, CoQ10H_2_, idebenone, idebenol, QS10 and EPI-743.

CoQ10 is one of the supplements most commonly used by patients with mitochondrial diseases (>40%) [46]. Apart from being the mandatory cofactor of complex I, CoQ10 is a scavenger of ROS, an inhibitor of lipid peroxidation, and an overall inhibitor of oxidative stress [47]. The pleiotropic role of CoQ10 in protecting mitochondria and its benefits regarding neurological diseases such as Alzheimer’s and Parkinson’s, MS, depression, and others have been reviewed elsewhere recently [48]. With respect to LHON, CoQ10 stands out among supplements because the interaction of its endogenous form with complex I is hindered by the mutations that cause LHON [11,49]. CoQ10 has been shown to protect retinal cells against oxidative stress, prevent RGC loss in cell or animal models of ON, and show a beneficial effect on glaucoma patients when associated with vitamin E [45]. However, there is no data documenting its role in LHON apart from one ancient case report of rapid visual recovery in one LHON patient after CoQ10 treatment [50]. One drawback of CoQ10 is its poor biodisponibility. CoQ10 has a chain of 10 isoprenoid repeats conjugated with a benzoquinone ring (Figure 4) and is, therefore, extremely lipophilic, with poor systemic availability and permeance in the blood-brain barrier, as well as high thermolability [51].

Another issue is that, in the context of LHON, with altered complex I and unchanged endogenous CoQ10 levels, it is uncertain whether exogenous CoQ10 can be effectively reduced to CoQ10H_2_ to play its role as a complex III cofactor and, in turn, restore ETC efficacy.

Recent studies revealed deficiencies in vitamin B12 [52] or in both taurine and vitamin B3 (nicotinamide) [53] in LHON patients. It has been proposed that vitamin B12 levels should be monitored regularly or, if necessary, corrected in LHON carriers [52]. Interestingly, vitamin B3 is the source of NAD/NADH for complex I, and supplementation with vitamin B3 has been shown to be protective against glaucoma by supporting mitochondrial health and metabolism in a mouse model [54]. Taurine is a sulfured amino acid mainly provided by food. It plays a key role in complex I function, in stabilizing the inner mitochondrial membrane gradient, and in regulating the balance between the oxidized and reduced NAD and oxidized and reduced glutathione [53].

In summary, while their safety does not appear to be an issue when used as recommended, these dietary supplements show promising molecular functions and cellular roles. However, their efficacy remains uncertain, and effective doses need to be investigated.

### 4.3. Exercise

Experimental or clinical studies have shown that physical activity can be protective in neurodegenerative, mitochondrial, or retinal diseases and associated with increased serum, brain, and/or retina levels of neurotrophins such as brain-derived neurotrophic factor (BDNF) [55]. Endurance training is known to provide patients with increased mitochondrial biogenesis and function, antioxidant enzyme activity, and maximal oxygen uptake, and the benefits are sustained over time [56]. Aerobic exercise was shown to delay RGC death after optic nerve injury in a rat model in a BDNF-dependent pattern [57]. As skeletal muscle function is not involved in LHON, a wide range of physical and sporting activities, such as walking, swimming, static workouts, indoor cycling, treadmills, or elliptical machines, are accessible and beneficial to patients. Many collective sports are also accessible to the visually impaired with the help of handi-sport associations. There is no doubt that physical activity or exercise should be recommended to LHON carriers. LHON patients should be advised to contact specialized associations to help them begin or help them play their chosen sport.

## 5. Environmental Risk Factors

### 5.1. Smoke, Smoking, and Vaping

An association with the smoking of tobacco has been observed for decades in patients converting to LHON [58]. Convergent studies revealed a strong and consistent association between LHON triggering and smoking in LHON carriers, regardless of gender and alcohol intake, leading to a clinical penetrance of 93% in men who smoked in one of the studies [16,59]. Tobacco smoking was associated with an increased risk of LHON triggering, and heavy smokers were also found to be more likely to be affected than light smokers (in relation to the maximum number of cigarettes consumed per day) [16]. Nicotine was shown to inhibit complex I and OXPHOS, increase ROS levels, reduce ATP generation, and favor mitochondrial fragmentation, although it also showed some paradoxical neuroprotective effects in neurodegenerative diseases [60]. Studies on a diversity of human cell or rodent models showed that cadmium [61], carbon monoxide and cyanides [62], and short-chain aldehydes [63], all present in cigarette smoke, impact OXPHOS and mitochondrial function negatively. Notably, heavy metal cadmium is a major threat to neurologic health, and the Agency for Toxic Substances and Disease Registry ranked it as the seventh most hazardous substance to human health [61]. Cadmium is widely used in industrial processes, contaminates soil and water, is absorbed by plants, and ultimately reaches the human body, where it accumulates throughout life. It has been shown that cadmium efficiently enters the nervous system with multiple neurotoxic outcomes and, in particular, impairs OXPHOS and increases ROS production [61]. The same combustion products may be present in the smoke of various origins, such as charcoal grills, car exhausts, flawed heaters, or faulty wood-burning stoves, which LHON carriers should also avoid direct exposure to, even though only a few articles have reported LHON conversion after exposure to inefficient wood-burning stoves, large rubber tire fires, or in firefighting workers [64,65].

Nicotine is also present in electronic nicotine dispensing systems (ENDS, also known as electronic cigarettes). Recent studies on cell or rodent models revealed that inhalation of ENDS vapor is far from being innocuous for mitochondrial health. Selenium and arsenic present in ENDS vapor was found to increase ROS production [66,67], and the reaction products between flavor aldehydes and e-liquid solvents (propylene glycol or glycerin) were reported to induce cytotoxicity and mitochondrial dysfunction [68].

### 5.2. Alcohol Abuse

An association with alcohol consumption has been observed for decades in patients converting to LHON [58]. A recent study reported that excessive alcohol intake at disease onset remained more frequent in male LHON patients than in healthy LHON carriers and the male general population [69]. A large-scale study suggested an association between increased risk of LHON triggering and alcohol only in patients with a heavy alcohol intake [16]. Alcohol neuropathogenicity has been known for many years [70], and the association of either acute or chronic ethanol exposure with caspase 3 activation, neuronal cell death, and neurodegeneration has been demonstrated in rodent models [71]. Mitochondria are the primary targets of chronic alcohol intake due to direct or indirect inhibition of complexes I and III and increased production of ROS and reactive nitrogen species (RNS) resulting from ethanol metabolism [72]. Furthermore, it has been shown that contamination of common alcoholic beverages with cadmium is not uncommon at concentrations of the 100-nM scale or more [73]. Accumulation of cadmium throughout life could, therefore, play a role in LHON in patients with a long history of excessive alcohol consumption.

Recent studies have led to a better understanding of alcohol neuropathogenic outcomes relevant to LHON. Alcohol was shown to upregulate NADPH oxidase 4 (NOX4) in mouse alveolar macrophages [74], and NOX4 was found to impede the expression of the five ETC complexes, therefore inhibiting mitochondrial respiration and ATP production, and inducing oxidative stress in human astrocytes grown in vitro [75]. Endoplasmic reticulum (ER) and mitochondria are physically and functionally linked by specialized microstructures called mitochondria-associated ER membranes (MAMs). MAMs finely regulate ER-mitochondria Ca^2+^ transfer and ER stress and are mandatory for mitochondrial homeostasis. Dysregulation of MAMS due to mutations within the wolframin gene *WSF1* causes Wolfram Syndrome, a severe mitochondrial disease including optic neuropathy, along with hearing impairment and diabetes [76]. A recent study using a mouse model of alcohol-associated liver disease revealed that alcohol increased the numbers of MAMs, resulting in mitochondrial dysfunction, in a pattern dependent upon the MAM-associated pyruvate dehydrogenase kinase 4 (Pdk4/PDK4) [77]. This finding echoes a previous metabolomic study that revealed specific ER stress in fibroblasts from LHON patients [78]. Together, these studies also indicate that NOX4, MAMs, or PDK4 could be plausible therapeutic targets for LHON.

### 5.3. Illicit Drug Abuse

The small size of the population, which is susceptible to both optic neuropathy and illicit drug abuse, does not make it any less of a public health issue, especially as the populations concerned may be far removed from care and screening pathways. Most of the psychoactive substances abused show outcomes comparable to alcohol in terms of mitochondrial function in neurons [79]. Few data are available about this issue regarding LHON yet. Illicit drugs impacting mitochondria in neurons are amphetamine, cocaine (with optic neuropathy presumably resulting from chronic orbital inflammatory disease after heavy intake), 3,4-methylenedioxy-methamphetamine (MDMA, ecstasy), synthetic opioids (fentanyl), or purposely inhaled solvents such as toluene [79,80].

### 5.4. Organic Solvents

Chemicals causing toxic optic neuropathy in healthy people are even more likely to threaten the optic nerve in LHON carriers. Occupational or incidental exposure to organic solvents is an unquestionable risk factor. Exposure to industrial toxins such as n-hexane and its neurotoxic metabolite 2,5-hexanedione (2,5-HD) or to polycyclic aromatic hydrocarbons has been reported to trigger LHON [14,81,82]. Methanol intoxication through occupational activities or consumption of adulterated alcohol can also result in toxic optic neuropathy, and it is assumed that methanol is converted to formate, which impedes OXPHOS by inhibiting complex IV of the ETC [83]. Occupational or purposeful inhalation of toluene (methyl benzene, found in many commonly used glues, paints, and industrial products) may also result in optic neuropathy [84,85,86].

### 5.5. ETC Inhibitors in Food

Given the complex I dysfunction in LHON, it is reasonable to speculate that inhibitors of ETC present in food might be risk factors, whether they are natural molecules or phytosanitary inputs. However, care must be taken with studies using high doses (often the ten micromolar scale or more) of a single molecule in cell models, even though these studies provide valuable molecular and cellular insight. In addition, singled-out treatment with an isolated molecule does not replicate the impact of eating the whole food it is from, as it rules out possible synergistic or antagonistic events. As a result, very few studies appear conclusive enough for reliable counseling. The case of the soursop, a tropical fruit popular in the southern hemisphere, is worth mentioning: on the one hand, sub-nanomolar doses of rolliniastatin-1, a complex I inhibitor naturally present in soursop, were found to impair oxygen consumption and increase the apoptosis rate of cybrid cells carrying the LHON mutation m.3460G>A as compared to wild type cybrids (cybrids are cell models whose mitochondria are replaced by mitochondria harboring a mutated genome) [87], but on the other, soursop as a whole contains proven neuroprotective tryptamine-derived alkaloids [88].

Succinate dehydrogenase (SDH) inhibitors (SDHIs) are among the most widely used pesticides worldwide to limit the proliferation of molds on plants and fruits. SDH is nothing less than complex II of the ETC and, as such, is the second source of reduced CoQ10 amenable to processing by complex III [27]. Thus, in the context of abnormal complex I functioning LHON carriers, concomitant inhibition of complex II may result in both accumulation of unreduced CoQ10, which could further increase oxidative stress, and limited supply of reduced CoQ10 to complex III, which could further hinder ETC. As a result, the mitochondrial dysfunction may be exacerbated. It has been proposed that SDHIs may be harmful to patients with mitochondrial diseases since two SDHBIs, bixafen and fluxapyroxad, were shown to cause apoptosis in cells from patients with Friedrich ataxia, a mitochondrial disease caused by mutations in the frataxin gene [89]. Nonetheless, no data related to optic neuropathy is available, and the demonstration that SDHIs are risk factors in LHON seems out of reach, given that they are somehow ubiquitous in food (and probably at very low concentrations) and are associated with a myriad of confounding factors in real life.

### 5.6. Quaternary Ammonium Salts

Quaternary ammonium salts (“quats”) are salts of quaternary ammonium cations combined with a negatively charged anion. They are widely used as disinfectants, surfactants, fabric softeners, and antistatic agents in a professional context but also in personal life (shampoo, detergents, preservatives, etc.). Exposure to quaternary ammonium salts is high and almost uninterrupted throughout life, and it is no exaggeration to write that our environment is “bathed in quats”. Benzalkonium chloride (BAK) is the most commonly used, present in eye drops, nasal sprays, and cosmetics. The adverse effects of BAK on mitochondrial function have been known for many years [90], and recent in vitro studies shed new light on complex I inhibition by BAK [91,92]. More specifically, BAK was also shown to inhibit complex I and reduce ATP generation at pharmacological concentrations in human cybrid cells with the m.11778G>A LHON mutation, as compared to the control [93].

## 6. Drugs

### 6.1. Authorized or Candidate Therapeutic Drugs for LHON

#### 6.1.1. Idebenone

**Figure 5:**
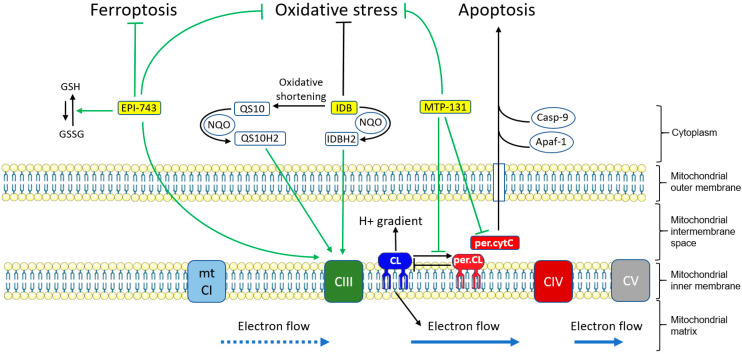
Schematic representation of the main mechanisms of idebenone, EPI-743, and elamipretide. EPI-743 can reduce oxidative stress and ferroptosis, particularly by supporting the conversion of oxidized glutathione (GSSG) to its reduced form (GSH). IDB can be converted to QS10 by metabolism (oxidative shortening). Idebenone (IDB) and QS10 can be reduced by NQOs (NQO) to idebenol (IDBH2) and QS10H2, respectively. IDBH2, QS10H2, and EPI-743 can bypass mutated complex I (mt CI) and thus relaunch electron transfer to and from complex III (CIII). Elamipretide (MTP-131) inhibits the conversion of cardiolin (CL) and cytochrome c to their peroxidated forms (per.CL and per.cytC, respectively). This preserves CL function and prevents the release of cytochrome c into the cytoplasm, where it could induce apoptosis upon binding to apoptotic protease activating factor 1 (Apaf-1) and caspase-9 (Casp9). Electron flow is depicted as blue arrows (solid: normal, dotted: defective). CIV: complex IV, CV: complex V.

Idebenone (IDB) is the only approved drug for LHON so far (European Medicine Agency). An international conference held in 2016 has provided consensus statements for the therapeutic management of LHON with IDB [94]. IDB (2,3-dimethoxy-5-methy-6-(10-hydroxy)decyl-1,4-benzoquinone) is a synthetic CoQ10 analog designed to overcome low CoQ10 bioavailability while maintaining or improving its benefits on mitochondrial function [95]. Structurally, in idebenone, the 50-carbon isoprenoid chain of CoQ10 is substituted by a 10-carbon aliphatic chain with a terminal hydroxyl group (Figure 4). NAD(P)H: quinone oxidoreductases (NQOs) are enzymes which catalyze the reduction of short-chain quinones into hydroxyquinones, with hydroxyl groups replacing ketones. IDB was shown to be efficiently reduced to idebenol by NQOs, and biochemical studies have revealed that idebenol is an effective substrate for complex III [95]. For this reason, idebenol is thought to bypass complex I, provide electrons for complex III, and restore ETC, in addition to other beneficial functions, including being an antioxidant [96] (Figure 5).

Interestingly, although rarely mentioned, IDB has also been shown to be an activating ligand of PPARγ, a nuclear receptor with a pleiotropic role, including limiting inflammation and oxidative stress and enhancing mitochondrial biogenesis [97]. IDB is readily metabolized through oxidation to a range of other short-chain quinones, including 6-(9-carboxynonyl)-2,3-dimethoxy-5-methyl-1,4-benzoquinone (QS10), where the terminal hydroxyl group is replaced by a carboxylic group (Figure 4). QS10 is also a substrate for NQOs and has been found more efficient than IDB for restoring OXPHOS in human cell or zebrafish models [98] (Figure 5).

After the first report of the rapid improvement of an LHON patient after IDB treatment as early as 1992 [99] and after other case reports, a prospective, placebo-controlled study with 85 LHON patients [100] and two retrospective analyses of cohorts of 103 or 111 LHON patients [8,101] convergently showed that IDB stabilized or improved visual function in approximatively 40–45% of subjects, albeit to variable extents, as compared to the untreated patients. Patients with the mutation m.3460G>A appeared to respond to the treatment less frequently than others [101]. Early and prolonged IDB treatment in patients seemed to be most favorable for improvement. No major adverse events were reported. These important studies have been confirmed by the recent retrospective investigation of a cohort of 72 Dutch LHON patients, 53% of whom showed improvement with IDB treatment [102]. It is important to note that the statistical power of such studies may be impeded by issues such as the relative rarity of the two mutations m.3460G>A and m.14484T>C (as compared to m.11778G>A, which accounted for 60–75% of the patients in these studies), together with the apparently low response ratio of m.3460G>A patients and the occurrence of rare spontaneous recovery of m.14484T>C patients. There was also considerable variability in the age of the patients, in the time between disease onset and treatment, and the duration of the treatment. Improved visual acuity in LHON patients treated with IDB late after onset (>5 years) was recently reported, suggesting that dysfunctional RGCs may survive the acute phase and retain their ability to recover function in the long term [103]. Further studies are therefore expected to provide better insight into IDB treatment in LHON.

#### 6.1.2. EPI-743

Like idebenone, EPI-743 (α-tocotrienol quinone or vatiquinone) is a CoQ10 synthetic analog designed to overcome low bioavailability [104]. EPI-743 is much more hydrophilic than CoQ10 due to its additional hydroxyl groups and shortened isoprenic chain (Figure 4). EPI-743 increases glutathione biosynthesis and is both a cofactor and a substrate for NQO1 [105] (Figure 5). EPI-743 is amenable to reduction into α-tocotrienol dihydroquinone by quinone reductase, resulting in its two ketone groups being replaced with two hydroxyl groups, making it an even stronger electron donor [106]. Nevertheless, the ability of α-tocotrienol dihydroquinone to transfer electrons to complex III as IDB does has not yet been formally demonstrated.

EPI-743 treatment was beneficial to patients with rare inherited neurodegenerative mitochondrial disorders, namely mitochondrial encephalomyopathy with lactic acidosis and stroke-like episodes (MELAS) or Leigh syndromes or Friedreich’s ataxia [104,105,107,108]. Notably, EPI-743 improved the brain redox status of patients, as shown with single photon emission computed tomography (SPECT) imaging [104]. EPI-743 thus appeared to be a good candidate drug for LHON. Two studies have shown that oral treatment of LHON patients with EPI-743 resulted in improved visual acuity, visual fields, color vision, and/or quality of life (four in five patients and one in two patients, respectively) in the absence of adverse events [109,110], but no further investigation on EPI-743 and LHON has been published since. Notably, EPI-743 has since been shown to prevent ferroptosis through its positive outcome on reduced glutathione levels [111]. Since several very recent and convergent studies have disclosed that ferroptosis inhibition is beneficial to RGC survival in experimental optic neuropathies or glaucoma [112,113,114,115], EPI-743 could soon be the focus of renewed interest.

#### 6.1.3. Elamipretide

Elamipretide (or MTP-131) is a small water-soluble tetrapeptide, permeant to the brain-blood barrier, readily absorbed by neural cells, and targeting the inner mitochondrial membrane where the ETC components are found [116]. A wealth of in vitro, preclinical, or clinical studies revealed its impressive potential for mitochondrial function and neuronal survival: enhanced mitochondrial respiration, activated neural mitochondrial biogenesis, enhanced mitochondrial fusion, inhibited mitochondrial fission, increased mitophagy, attenuated mitochondria-derived oxidant levels, and neural oxidative stress, decreased neuroinflammation, and increased cellular ATP production thanks to improved ETC [116,117]. Notably, elamipretide has been shown to inhibit peroxidation of mitochondrial cardiolipin (CL) and cytochrome c [117]. CL is a phospholipid of the inner mitochondrial membrane that binds cytochrome c and plays a key role in both ETC and maintenance of the proton gradient. Peroxidation of CL and cytochrome c as a result of oxidative stress may result in the loss of CL function and the release of cytochrome c into the cytoplasm, where it may induce apoptosis on binding to the apoptotic protease activating factor 1 (Apaf-1) and caspase-9 (Casp9). Therefore, restoring CL and cytochrome c functions with elamipretide improves electron transfer from complex III to complex IV and subsequently OXPHOS, as well as preventing apoptosis from cytochrome c (Figure 5).

Elamipretide was recently reported to be well tolerated in patients with mitochondrial myopathy [118]. Elamipretide associated with a TNF-α inhibitor (etanercept) improved RGC survival in a mouse model of traumatic ON [119]. Together, these data made elamipretide an obvious candidate for LHON. A recent study reported improved visual function, color discrimination, and contrast sensitivity with topical elamipretide treatment in 12 LHON patients, with no severe adverse events [120]. Given that this treatment was initiated long after vision loss, at a stage when recovery is extremely rare, these findings are particularly promising.

### 6.2. Other Drugs with Possibly Adverse Outcomes on the Optic Nerve

Drugs likely to injure the optic nerve of “healthy” subjects can be expected to be a threat to LHON carriers. A recent international consensus has provided wide-ranging and valuable insight into the clinical guidance for prescribing drugs for patients with primary mitochondrial diseases [121].

Mitochondrial toxicity has been recognized for many years as a complication of antiretroviral therapy with nucleoside analogs. Nucleoside analogs, and especially zidovudine (AZT), are prone to hindering OXPHOS and inhibiting mitochondrial DNA replication and function, leading to neuropathies [122]. In a few cases, LHON triggering in previously unaffected carriers was reported subsequent to, and likely caused by, the initiation of highly active antiretroviral therapy (HAART) to manage human immunodeficiency virus (HIV) infection [123,124,125,126,127]. Strikingly, in some patients with the m.14484T>C mutation, the visual impairment was far more severe than expected with this mutation and included short- or long-term loss of light perception [123].

The anti-tuberculosis antibiotic ethambutol has been shown to cause toxic ON in 100,000 cases each year [128]. Ethambitol is a metal chelator and, as such, may inhibit the ETC, which relies on the activity of iron-binding complex I and copper-binding complexes III and IV. Ethambutol was also found to raise ROS levels in RGCs in vitro and in animal models [128]. Other antibiotics (isoniazid, another anti-tuberculosis drug, linezolid, aminoglycosides, or fluoroquinolones such as ciprofloxacin) have also been reported to result in ON, although full visual recovery occurred after discontinuation of the medication [128].

Recently, the anti-epileptic drug sulthiame, an inhibitor of carbonic anhydrase, was reported to trigger LHON in two unrelated previously healthy carriers and was shown to impair the respiration of fibroblasts from LHON donors in vitro [129]. Interestingly, another carbonic anhydrase inhibitor prescribed for epilepsy, topiramate, had been reported to have triggered LHON in a previously healthy carrier [130]. It has been speculated that carbonic anhydrase inhibitors may share a common pathogenic basis through OXPHOS impairment due to bicarbonate ion deprivation of specific mitochondrial enzymes [129].

Reports of LHON conversion due to other medications are very rare and even sometimes anecdotal. Anesthetics, and in particular propofol (2,6-diisopropylphenol), which alters complex I activity, may induce neurodegeneration through OXPHOS inhibition [131]. In a consensus statement from the USA Mitochondrial Medicine Society, patients with mitochondrial diseases are mentioned as being at an increased risk of anesthesia-related complications; on the other hand, they mention anesthetics as generally safe given that pre-operation fasting is limited and an energy source is provided during anesthesia [56]. Phosphodiesterase 5 (PDE5) inhibitors, commonly used in the treatment of erectile dysfunction, may cause ophthalmic complications, mainly non-arteritic ischemic optic neuropathy (NAION) [132], but only one case of triggering of LHON as a result of using a PDE5 inhibitor, has been reported, which is of very little significance compared to the millions of users worldwide [133]. Erythromycin has been reported to have triggered LHON in one single case to date [90]. The anti-epileptic drugs tiagabine and vigabatrin induced toxic ON in a few cases [134]. The immunosuppressant tacrolimus has also been reported to cause optic neuropathy on rare occasions [135].

## 7. Discussion

Here, we present an updated review of the environmental determinants of the onset and expression of LHON. We also review current knowledge of idebenone and candidate therapeutic drugs, as well as drugs with a possibly negative impact on LHON carriers. We draw attention to the fact that this review is an off-label discussion with no claimed recommendation for medical practice.

LHON is a devastating vision disease caused by degeneration of the RGCs and optic nerves with incomplete penetrance and unpredictable time of onset throughout life. Recent revision of the frequency of the mutations that cause LHON in the general population (1/800–1000) suggests that the population with LHON susceptibility is much larger than previously thought [13,17]. Previous reviews predominantly focused on drugs and did not encompass the wealth of environmental factors with a possible impact on LHON onset, whether they are protective or risk factors.

**Figure 6:**
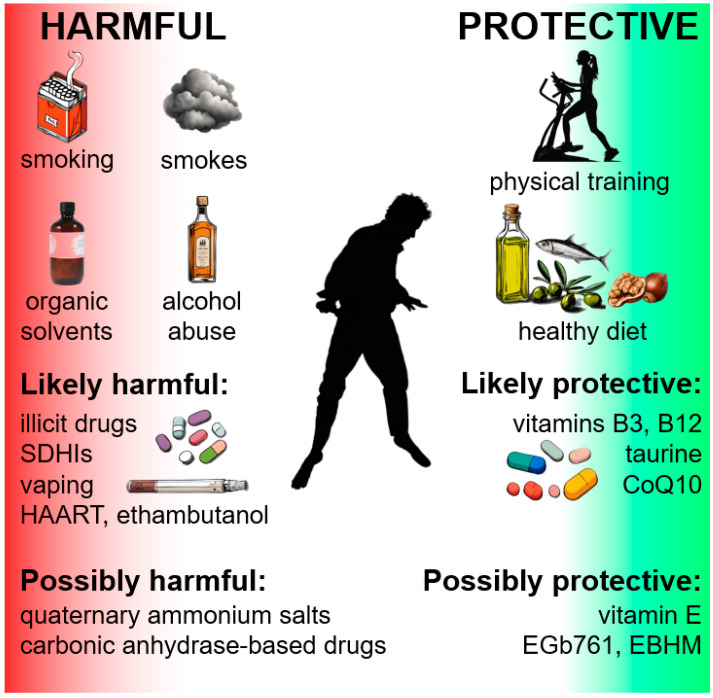
Graphic summary of the main environmental factors known or assumed to be protective or harmful for LHON carriers.

It appears that lifestyle is critical for LHON mutation carriers, who need a healthy diet (possibly ketogenic or Mediterranean) and physical exercise, and who should exclude smoking, binge drinking, and exposure to smoke and organic solvents (Figure 6).

It is more difficult to conclude the role of dietary supplements, although their characteristics suggest that they should be beneficial. Interestingly, the natural form of EPI-743, α-tocotrienol quinone, is a metabolite of α-tocotrienol, a lipid in the vitamin E family. It has been suggested that dietary vitamin E could be a source of α-tocotrienol quinone/EPI-743 [106]. The impact of common, “everyday” chemicals (phytosanitary products and quaternary ammonium salts) remains unclear, although exposure to them could be minimized as a matter of precaution. Very few drugs seem to represent a threat to LHON carriers. The issue raised by ethambutanol can be resolved by anti-tuberculosis vaccination. Basically, reports of LHON triggered by adverse drug reactions must be viewed with great caution so as not to deprive subjects of necessary medication. With regard to therapeutic drugs for LHON, promising ongoing studies will provide new insights concerning the development and efficacy of gene therapy and idebenone, as well as candidates such as elamipretide, or new molecules, such as recently identified naphthoquinone derivates [136].

Apart from environmental factors, secondary genetic determinants also play a critical role in LHON expression. In cis, the haplogroup (the nucleotide “background” of mtDNA) is known to be a key determinant of LHON onset. LHON is triggered more frequently in individuals with both the m.11778G>A mutation and mtDNA haplogroup J and the odds of vision loss were assessed in a recent study as being almost three times greater in LHON carriers with haplogroup J compared to their counterparts with haplogroup H [14]. The haplogroup can exacerbate the sensitivity of LHON carriers to environmental factors, as shown for haplogroup J with regard to 2,5-hexanedione [82]. In trans, the alleles 157C>T (53R>W) and 572G>T (191G>V) in the nuclear genes *PRICKLE3* and *YARS2*, respectively, have been shown to be secondary factors of susceptibility for LHON [137,138]. As these two genes are in chromosome X, they may also account for the sex bias observed in LHON expression.

Novel environmental factors are expected to be investigated in the short term. The psychosociological context may play a role in the onset of LHON. It has been reported that more than 10% of LHON patients had pre-existing mood disorders before vision loss [139]. The long and worrisome diagnosis odyssey often results in a delay in therapeutic care, as well as severe psychological outcomes [140].

In conclusion, the diversity of environmental factors underlies the incomplete penetrance and variability of LHON expression. Altogether, these data underline the importance of multidisciplinary management of LHON patients and carriers.
